# The Telephone in Medical Practice

**Published:** 1915-02-13

**Authors:** 


### The Hospital

The Workers' Newspaper of Administrative Medicine and Institutional Life, Administration, National Insurance and Health.

No. 1495, Vol. LVII. SATURDAY, FEBRUARY 13, 1915. PRICE ONE PENNY.

### THE TELEPHONE IN MEDICAL PRACTICE.

An incident related a few days ago in the newspapers contains an impressive warning of the risks involved in the use of the telephone as a medium for the communication of medical directions. A medical practitioner ordered the preparation of a solution of eserine sulphate, and sent the directions by telephone. The dispenser, mistaking the figures, made the solution much stronger than the doctor had intended, with the result that the administration of the medicine to the patient was followed promptly by a fatal result. In this individual instance there was some mitigation of the position in the fact that the patient was the subject of a malignant growth, and was therefore beyond the reach of hope. This, however, was an accident of the position and not its essence. The brief fact is that a poisonous dose of medicine was administered because the directions for the compounding of the medicine were conveyed through the telephone. The moral is an obvious one, and we should be glad to learn that the profession had adopted a rule against the cultivation of this practice. Even if it be allowed that a medical man will generally be so explicit and careful that an error is unlikely, some degree of risk remains in the very nature of things, as indeed the above-mentioned incident shows. Further, the example of the doctor is apt to be followed by the nurse or other person who is not so conscious either of individual responsibility or of the need for care. The only safe rule, and it ought to be enforced in all hospitals and nursing institutions, is that the prescribing and ordering of medicines should be by written directions, and that for such purposes the telephone should be forbidden. Everyone with experience knows that the interpretation of sounds received through the telephone depends very largely upon their setting and context, and thus quantities and figures, where the context is not helpful, frequently come to grief. Even the expert ears of the exchanges not infrequently fall into error, and so inflict upon the long-suffering subscriber the answering cry of "Wrong number."

The particular rule here commended may be advised on more general grounds. Useful and even necessary as the telephone is, it is often employed in a manner particularly obnoxious to members of the medical profession. Only too often does the practitioner find himself "rung up" to answer some trivial and wholly unnecessary question; or his opinion is requested in circumstances where the implication is plain that he is expected to give advice gratuitously. Again, professional work is of a character in which calls of this order are specially irritating and inconvenient. Among the minor ills of life, what is more likely to freeze "the genial current of the soul" than an imperative demand to answer the telephone when engaged in a critical and responsible medical examination? It follows that the interest of medical practitioners is to reduce to a minimum the occasions on which the piercing tintinnabulation of this insistent instrument is set in operation. But if doctors would avoid this metallic persecution they must, so far as in them lies, be careful to protect others from its horrid and ear-splitting vibration. Here is an opportunity. In ninety-nine cases out of a hundred medicines can be ordered by the written word. Let this be employed, and let the use of the telephone for such a purpose be condemned. By so acting medical men will not only follow the pathway of safety, but they will be proclaiming to nurses and patients and friends that what is good to meet an emergency ought not to be employed for lesser events or as a convenient shelter for habitual laziness.

The issue may be presented in a still broader aspect. At present there is no etiquette of the telephone—or none, at least, outside the exchanges—and one is badly needed. Especially is it needed in the interests of the medical practitioner. The sender of a message chooses his own time, which, though convenient to himself, may be highly inconvenient to the subscriber at the other end of the wire; and this inconvenience, as we have already pointed out, is especially trying to anyone engaged in medical work. We would therefore have as a first rule, that no communication should be made by telephone which can possibly be made by letter. Again, the arrangement of the telephone is such that the caller practically walks into his victim's house, or even into his consulting room, whether with or without permission. The question is not, Is Mr. So-and-So engaged? but briefly, Are you there? Working or playing, eating or sleeping, dressing or bathing, there is no escape. Here, then, comes the opportunity for Rule No. 2—namely, that prior to an attempted conversation the calling subscriber should politely ask, Is Dr. or Mr. So-and-So engaged? Were these two rules generally adopted, the telephone would be regarded with a more kindly eye and would cease to receive those terms of abuse which respect for our readers forbids us to register on the printed page. Had we a free hand we would enforce still a third rule, to the effect that the telephone should not be used for prolonged conversations. To the neglect of this, one of the sexes—prudence forbids us to be more precise—seems to be particularly prone, and possibly some mitigation of the evil might be secured were every instrument to carry the injunction of the Preacher, "Let thy words be few." Here, possibly, we urge a counsel of perfection. But we shall surely carry the sympathy of our readers in the conclusion that the abuse of the telephone is increasing, and that it ought to be abated.

